# Differences in cell subsets between sun-exposed and unexposed skin: preliminary single-cell sequencing and biological analysis from a single case

**DOI:** 10.3389/fmed.2024.1453940

**Published:** 2024-10-29

**Authors:** Feng Zhou, Yu Sun, Xinling Chen, Wenyi Hou, Jing Shen, Wei Lai, Kai Han, Yue Zheng

**Affiliations:** ^1^Department of Dermatology, Nanfang Hospital, Southern Medical University, Guangzhou, China; ^2^Department of Dermato-venereology, The Third Affiliated Hospital of Sun Yat-sen University, Guangzhou, China

**Keywords:** UV, skin, aging, photoaging, single-cell sequencing

## Abstract

**Introduction:**

The composition and subsets of skin cells continuously change in a dynamic manner. However, the specific microcosmic alterations of human photoaged skin, independent of chronologic aging, remain unclear and have been infrequently analyzed. This study aimed to evaluate the biological processes and mechanisms underlying cell-subgroup alterations in skin photoaging.

**Methods:**

We utilized single-cell sequencing and biological analysis from a single case to investigate the effects of photoaging. Skin punch biopsies were taken from sun-exposed forearm skin and unexposed buttock skin from the same individual for comparative analysis.

**Results:**

Our analysis identified 25 cell clusters and 12 skin cell types, revealing significant changes in unique gene expressions between the sun-exposed and unexposed skin samples. A comparison of cell numbers within each cluster revealed 9 dominant cell clusters in sun-exposed skin and 16 dominant cell clusters in unexposed skin. Enrichment analysis indicated that PD-L1 expression and the PD-1 checkpoint pathway were more prominent in sun-exposed skin, while MAPK, TNF-alpha, TGF-beta, and apoptosis pathways were more enriched in hair follicle cells of sun-exposed skin.

**Discussion:**

This study reveals changes in cell components in photoaged skin from a single case and provides novel insights into cellular subpopulations and pathology during repeated UVA-induced skin damage. These findings enhance our understanding of the complex interplay between different cells in photoaged skin and offer potential targets for preventing human skin photoaging and UV-induced skin cancers.

## 1 Introduction

The composition of skin cell subsets can be altered by both extrinsic and intrinsic factors (1).

In chronologically aged skin, studies have documented a reduction in cellularity within the epidermis, characterized by decreased numbers of melanocytes, mast cells, and Langerhans cells (2). While the dermis undergoes atrophy, there is a reduction in the number of fibroblasts and a decrease in subdermal adipose tissue (3). One of the primary causes of skin aging, known as photoaging, is induced by chronic ultraviolet (UV) irradiation.

Skin photoaging, one of the main causes of skin aging, is induced by chronic ultraviolet (UV) irradiation. UV radiation, particularly UVA (320–400 nm) and UVB (290–320 nm), penetrates the skin and induces a cascade of molecular events leading to skin damage. UVA radiation penetrates deeper into the dermis, leading to oxidative stress and damage to collagen fibers (4, 5), while UVB radiation primarily affects the epidermis, causing direct DNA damage and the formation of thymine dimers (6). The cumulative effects of these radiations result in characteristic changes associated with photoaged skin, including wrinkling, loss of elasticity, pigmentation changes, and an increased risk of skin cancer (7).

Photoaging involves not only molecular and structural changes but also alterations in various cell populations within the skin. Photodamaged skin frequently exhibits an increased number of hyperplastic fibroblasts, as well as an influx of inflammatory cells, including mast cells, histiocytes, and other mononuclear cells (8).

In addition, UV radiation negatively correlates with capillary density and the pericyte-to-endothelial cell (PC/EC) ratio in capillaries or venules, attributed to both relative and absolute loss of pericytes (9). However, the specific microcosmic changes in human photoaging skin, independent of chronologic aging, remain largely unexplored and poorly understood.

Conventional bulk RNA sequencing, which provides a view of averaged gene expression across cell populations, falls short in explaining the heterogeneity and plasticity of human epidermal cells. In contrast, single-cell transcriptomics has emerged as a highly effective approach for defining distinct cell populations. Single-cell RNA sequencing (scRNA-seq) technologies offer unprecedented opportunities to explore physiological and pathological transcriptomic changes at single-cell resolution. This advanced methodology allows for the identification of changes within various cell populations in specific tissues.

In this study, we conducted a comparative analysis between sun-exposed forearm skin and unexposed buttock skin from the same individual. By employing single-cell sequencing and biological analysis, we aimed to determine the biological processes and mechanisms driving changes in cell subgroups associated with skin photoaging. This approach enables a more nuanced understanding of how specific skin cell populations are affected by UV exposure, contributing to a deeper comprehension of the cellular and molecular underpinnings of photoaging.

## 2 Materials and methods

### 2.1 Clinical samples

**Figure 1:**
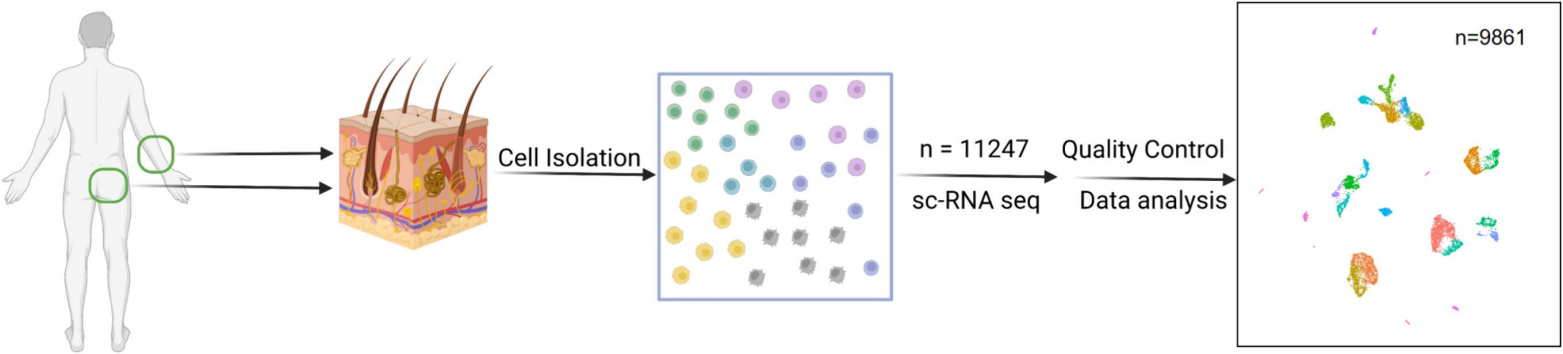
Flowchart overview of scRNA-seq of human forearm skin and buttock skin. This schematic was created using BioRender.com.

The forearm skin and buttock skin were obtained from the same healthy individual (Chinese woman, aged 54, Fitzpatrick skin type III) via skin punch biopsy (Figure 1). The samples were collected during the summer months to capture the effects of increased sunlight exposure. Skin surgeries were performed in a standardized surgical procedure. Then, 4-mm punch biopsies were obtained from healthy whole skin specimens from the forearm and buttock regions. Immediately after resecting the particular skin, the samples were placed in a MACS Tissue Storage Solution (Miltenyi Biotec, cat. no. 130–100-008) and kept for no longer than 1 h before being enzymatically and mechanically dissociated using the Whole Skin Dissociation Kit for human material (Miltenyi Biotec, cat. no. 130-101-540) and the gentleMACS Dissociator (Miltenyi Biotec), according to the manufacturer’s instructions. Cell suspensions were then filtered through 70-μm cell strainers (Falcon) and depleted of cell debris and dead cells using the Dead Cell Removal Kit (Miltenyi Biotec, cat. no. 130-090-101).

The subject underwent a comprehensive full-body skin examination conducted by a dermatologist before surgery, and medical records were thoroughly reviewed, focusing specifically on skin-related diseases and conditions that might affect the skin. There was no clinical evidence or history of inflammatory or systemic skin disease (e.g., systemic sclerosis and lupus erythematosus) or comorbidities that typically affect the skin and/or UV sensitivity of the skin (e.g., chronic immunosuppression and chronic renal failure). Furthermore, the patient did not have a history of UV therapy, exhibit clinical evidence of acute or chronic actinic skin damage, or present with tanned skin in the inguinoiliac region at the time of surgery. An independent set of skin samples was used for validation experiments.

The Medical Ethical Committee of the Third Affiliated Hospital of Sun Yat-sen University approved the study protocol (2021-331) and the informed consent form. The subject provided informed written consent.

### 2.2 Single-cell RNA sequencing

After enzymatically and mechanically disrupting the tissue, the samples were subjected to scRNA-seq using the 10X Genomics platform (v2 chemistry). This commercial version of the high-throughput Drop-seq protocol identifies cell populations by analyzing the expression of highly expressed genes in a high number of cells. After stringent cell filtration, 11,247 cells were retained for subsequent analyses.

Sequencing libraries were subsequently prepared following the Drop-seq methodology (56), using a Chromium Single Cell Controller and the v2 chemistry from 10X Genomics (cat. no. 120237). Thus, ~6,000 cells per sample were mixed with the retrotranscription reagents and pipetted into a Chip A Single Cell (10X Genomics, cat. no. 1000009), which also contained the Single Cell 3’ Gel Bead suspension and Partitioning Oil. The Chip was subsequently loaded into a Chromium Single Cell Controller (10X Genomics), where the cells were captured in nanoscale droplets containing both the reagents needed for reverse transcription and a gel bead. The resulting gel bead-in-emulsions (GEMs) were then transferred to a thermocycler to perform the retrotranscription, following the manufacturer’s protocol. Each gel bead contained a specific 10X Genomics barcode, an Illumina R1 sequence, a unique molecular identifier (UMI), and a poly-dT primer sequence. Therefore, from poly-adenylated mRNA, the reaction produced full-length cDNA with a unique barcode per cell and transcript, which allowed tracing back all cDNA coming from each individual cell. Following an amplification step, cDNA was further processed by fragmentation, end repair, and A-tailing double-sided size selection using AMPure XP beads (Beckman Coulter, cat. no. A63881). Finally, Illumina adaptors and a sample index (10X Genomics, cat. no. 120262) were added through PCR using a total number of cycles adjusted to the cDNA concentration. After sample indexing, the libraries were again subjected to double-sided size selection. Quantification of the libraries was carried out using the Qubit dsDNA HS Assay Kit (Life Technologies), and cDNA integrity was assessed using D1000 ScreenTapes (Agilent Technologies). Paired-end (26 + 74 bp) sequencing (100 cycles) was performed using a HiSeq 4,000 device (Illumina).

### 2.3 Data analysis

Raw sequencing data were processed using Cell Ranger, version 3.1.0, from 10X Genomics. For downstream analysis of the data, we used the Seurat package version 4.1.1 in R version 4.2.1. A total of 11,247 cells passed the quality control steps performed by Cell Ranger. To remove possible cell doublets, we filtered out cells with more than 99% UMI and less than 200 expressed genes. To remove potential apoptotic cells, we removed cells with more than 25% mitochondrial reads. The application of these filters resulted in a final dataset of 9,861 single-cell transcriptomes. To standardize cell clustering and visualize using Seurat, first, the data were scaled using the ScaleData function, and principal component analysis (PCA) dimensions were calculated using the RunPCA function. Next, unsupervised clustering of the data was performed using the FindNeighbors and FindClusters functions. For the FindNeighbors function, we used the first 30 PCA dimensions to construct a shared nearest neighbor (SNN) graph for our dataset. Then, we clustered the cells with the function FindClusters using a shared nearest neighbor (SNN) modularity optimization-based clustering algorithm with a resolution of 0.6. Finally, for visualization, we used the RunUMAP function with default parameters and 30 PCA dimensions. To identify genes with enriched expression in each cell cluster, we used the FindAllMarkers function in the integrated dataset. This function used the Wilcoxon rank-sum test to identify the representative genes of each cluster. These representative genes were used to establish the cell identity of each cluster, together with markers found in the literature for cell types typically present in the human skin. The average expression of a particular set of marker genes was used for cell type identification and was projected into UMAP or heatmap. Gene expression signatures were used for the definition of cell populations, as shown in Section 3.4. To infer sun exposure-related differences, we used the FindAllMarkers function to identify those genes whose expression is enriched in each cell cluster of the sun-exposed and unexposed skin datasets separately (only.pos = TRUE, logfc.threshold = 0.25). We used Gene Ontology (GO) analysis to enrich DEGs (logfc.threshold = 0) of fibroblast subclusters to explore their functions, and we also enriched genes (logfc.threshold = 0 except for macrophages/DC logfc.threshold = 0.1) obtained by the comparison of sun-exposed and unexposed groups of T cells, T-cell subcluster 1, fibroblasts, fibroblasts subclusters, mast cells, macrophages/DC, and sebaceous gland cells through GSEA to find enriched pathways in the sun-exposed groups. In addition, Kyoto Encyclopedia of Genes and Genomes (KEGG) analysis was used to enrich differentially expressed genes (DEGs) (Log2FoldChange > 0.25) of hair follicle cells between the sun-exposed and unexposed groups. We used the subset function to extract T cells and set the resolution to 0.4 for dimensionality reduction analysis clustering and further analysis.

## 3 Results

### 3.1 scRNA-seq analysis of sun-protected and exposed human skin

**Figure 2:**
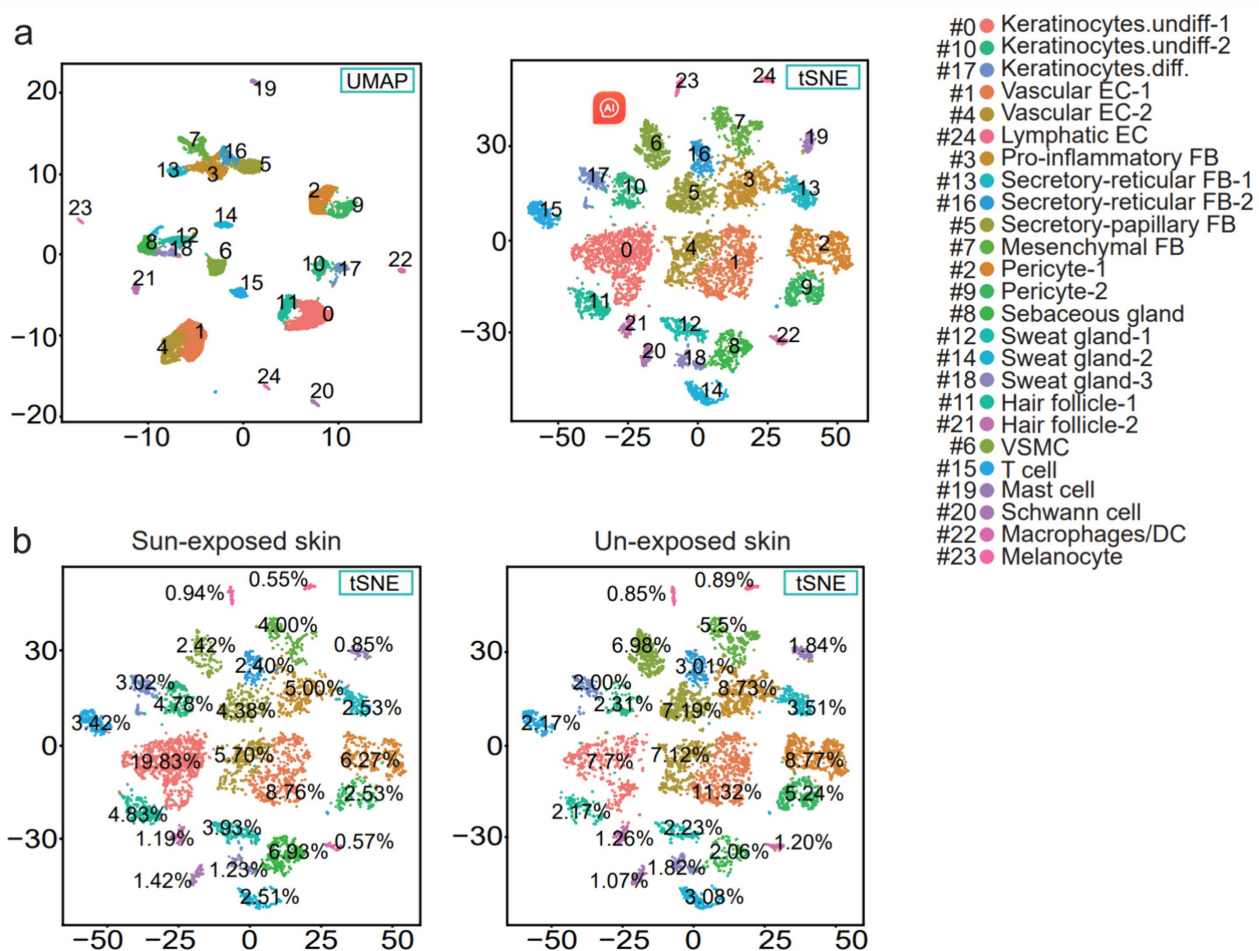
(a) UMAP and t-SNE plot depicting single-cell transcriptomes from sample human skin. Each dot represents a single cell (n = 9,861). Coloring is according to the unsupervised clustering performed by Seurat. SMC, vascular smooth muscle cell; EC, endothelial cell; DC, dendritic cells; FB, fibroblasts; Keratinocytes.undiff., undifferentiated keratinocytes; Keratinocytes.diff., differentiated keratinocytes. (b) t-SNE plot showing the percentage of the cell numbers in sun-exposed skin derived from the forearm skin sample and the percentage of the cell numbers in the unexposed skin from the buttock skin sample for the 25 clusters.

The cell suspensions obtained from the dissociation and filtration of forearm and buttock skin yielded a total of 9,861 cells, which were chosen for further analysis after quality control and data assessment (Figure 1). The human skin cell populations were visualized using uniform manifold approximation and projection (UMAP) and t-distributed stochastic neighbor embedding (t-SNE) (Figure 2a).

### 3.2 Differentially expressed genes (DEGs) in each cell cluster

**Figure 3:**
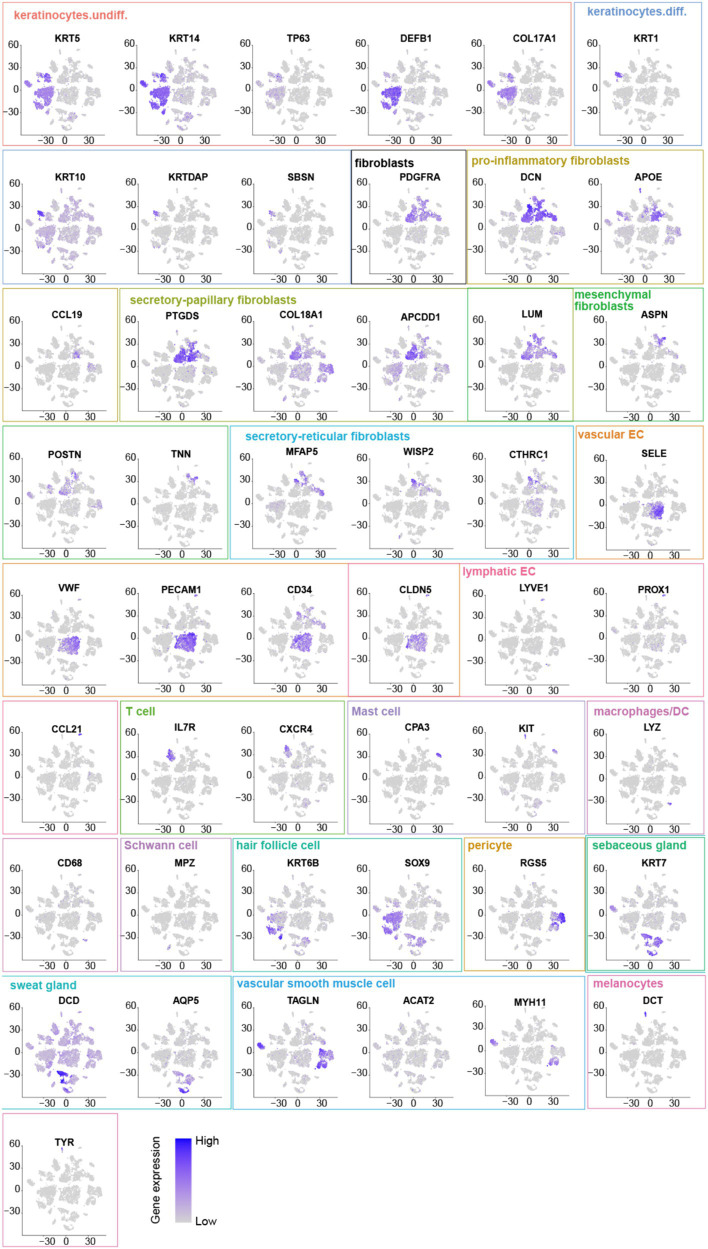
Marker genes: Undifferentiated keratinocytes [DEFB1, COL17A1, KRT5, KRT14, and TP63]. Differentiated keratinocytes [KRT1, KRT10, KRTDAP, and SBSN]. Fibroblasts [PDGFRA] include pro-inflammatory fibroblasts [DCN, APOE, and CCL19], secretory-papillary fibroblasts [LUM, COL18A1, APCDD1, and PTGDS], mesenchymal fibroblasts [LUM, ASPNPOSTN, and TNN], and secretory-reticular fibroblasts [MFAP5, WISP2, and CTHRC1]. Vascular EC [SELE, VWF, PECAM1, CD34, and CLDN5]. Lymphatic EC [CLDN5, LYVE1, PROX1, and CCL21]. T cell [IL7R and CXCR4]. Mast cell [CPA3 and KIT]. Macrophages/DC [LYZ and CD68]. Hair follicle cells [KRT6B and SOX9]. Pericyte [RGS5]. Sebaceous [KRT7] and sweat gland cells [KRT7, DCD, and APQ5]. Vascular smooth muscle cells (VSMC) [TAGLN, MYH11, and ACTA2]. Schwann cells [MPZ]. Melanocytes (#23 [DCT and TYR]).

**Figure 4:**
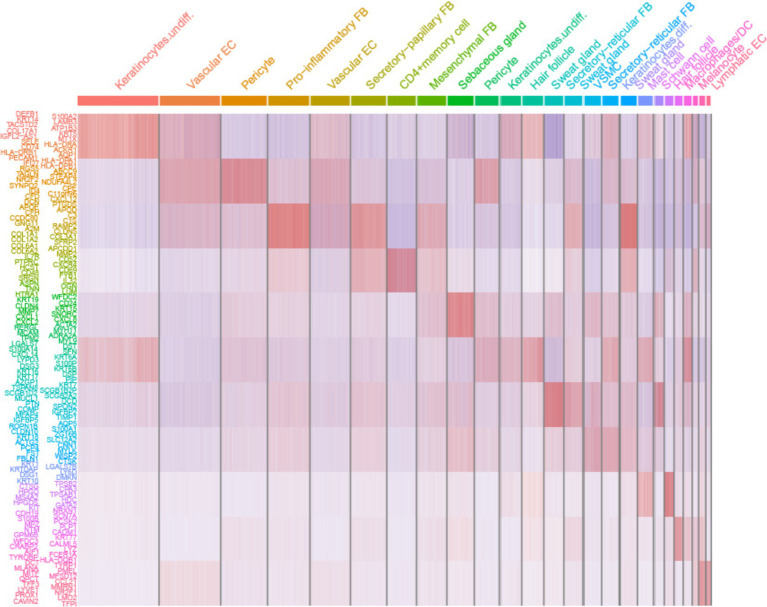
Heatmap showing the 10 most differentially expressed genes (DEGs) of each cell cluster, as determined by Seurat. Each column represents a single cell, and each row represents an individual gene. Overall, 10 marker genes per cluster are shown on the left. Red indicates maximum gene expression, and blue indicates no expression in scaled log-normalized UMI counts.

Comparing known markers with the most representative expressed genes of each cluster (Figures 3, 4) revealed the identity of the 25 cell clusters, all of which are known constituents of the human skin and represent 12 main cell types (Figure 2a). Among these clusters, seven comprised the two key cell types of the skin, keratinocytes and fibroblasts. Keratinocytes were detected in three clusters (#0, #10, and #17) which consist of undifferentiated keratinocytes (#0[DEFB1, COL17A1, KRT5, KRT14] and #10[KRT5, KRT14, TP63, COL17A1]) and differentiated keratinocytes (#17[KRT1, KRT10, KRTDAP, SBSN]) (10). Fibroblasts were found in five clusters (#3, #5, #7, #13, and #16 [PDGFRA]), including pro-inflammatory fibroblast (#3[DCN, APOE, and CCL19]), secretory-papillary fibroblast (#5[LUM, COL18A1, APCDD1, and PTGDS]), mesenchymal fibroblast (#7[LUM, ASPN, POSTN, and TNN]), and secretory-reticular fibroblast (#13[MFAP5] and #16[WISP2, and CTHRC1]) according to Llorenç Solé-Boldo’s research (10). Other identified cell clusters include endothelial cell (#1, #4, and #24), comprising vascular EC (#1 [SELE, VWF, and PECAM1] and #4 [PECAM1, CD34, and CLDN5]) and lymphatic EC (#24 [CLDN5, LYVE1, PROX1, and CCL21]) (10–13), immune cells (#6, #19, and #22) containing T cell (#6[IL7R and CXCR4]) (14), mast cell (#19[CPA3 and KIT]) (15, 16), and macrophages/DC (#22[LYZ, and CD68]) (10), hair follicle cells (#11 and #21[KRT6B and SOX9]) (15, 17–19), pericytes (#2 and #9[RGS5]) (17), sebaceous gland (#8 [KRT7]) and sweat gland cells (#12 and #18 [KRT7 and DCD] and #14[APQ5 and KRT7]) (20–23), vascular smooth muscle cell (VSMC: #15 [TAGLN, MYH11, and ACTA2]) (10, 15, 24), Schwann cell (#20[MPZ]) (25), and melanocyte (#23 [TYR and DCT]) (10). The marker genes for these clusters are shown in Figure 3 with a list of the complete names of the abbreviated genes, as shown in Table 1. In addition, the top 10 differentially expressed genes (DEGs) in each cluster are depicted in the heatmap (Figure 4; Table 2).

**Table 1:** Complete names of the abbreviated genes.

Gene	Full name	Gene	Full name
DEFB1	Defensin beta 1	APCDD1	APC downregulated 1
COL17A1	Collagen type XVII alpha 1 chain	PTGDS	Prostaglandin D2 synthase
KRT5	Keratin 5	ASPN	Asporin
KRT14	Keratin 14	POSTN	Periostin
TP63	transformation-related protein 63 and keratinocyte transcription factor KET	TNN	Tenascin N
KRT1	Keratin 1	MFAP5	Microfibril-associated protein 5
KRT10	Keratin 10	WISP2	WNT1-inducible signaling pathway protein 2
KRTDAP	Keratinocyte differentiation-associated protein	CTHRC1	Collagen triple helix repeat containing 1
SBSN	Suprabasin	SELE	Selectin E
PDGFRA	Platelet-derived growth factor receptor alpha	VWF	Von Willebrand factor
DCN	Decorin	PECAM1	Platelet and endothelial cell adhesion molecule 1
APOE	Apolipoprotein E	CD34	CD34 Molecule
CCL19	C-C motif chemokine ligand 19	CLDN5	Claudin 5
LUM	Lumican	LYVE1	Lymphatic vessel endothelial hyaluronan receptor 1
ITM2A	Integral membrane protein 2A	C3	Complement component 3
RARRES2	Retinoic acid receptor responder 2	HOXC10	Homeobox C10
BST2	Bone marrow stromal cell antigen 2	COMP	Cartilage oligomeric matrix protein
WIF1	Wnt inhibitory factor 1	MFAP5	Microfibrillar-associated protein 5
COL14A1	Collagen type XIV alpha 1 chain	CPE	Carboxypeptidase E
CLU	Clustering	PROX1	Prospero homeobox 1
COL18A1	Collagen type XVIII alpha 1 chain		

**Table 2:** Ten most differentially expressed genes (DEGs) in each cell cluster.

Cluster	Cluster annotation	The top 10 differentially expressed genes (DEGs)
0	Keratinocytes.undiff.-1	DEFB1, S100A2, KRT14, LAMB3, TACSTD2, ATP1B3, COL17A1, KRT5, IGFL2-AS1, and MT1X
1	Vascular EC-1	SELE, HLA-DRA, CD74, ACKR1, HLA-DRB1, AQP1, PECAM1, HLA-DPA1, IFI27, and HLA-DPB1
2	Pericyte-1	RGS5, ABCC9, TAGLN, STEAP4, NR2F2, NDUFA4L2, SYNPO2, CPE, PRRX1, and NOTCH3
3	Pro-inflammatory FB	CFD, CXCL12, DCN, PTGDS, APOE, APOD, CFH, C3, CCDC80, and C1S
4	Vascular EC-2	CD74, IFI27, HLA-DRA, HLA-DRB1, GNG11, RAMP2, A2M, PECAM1, CLDN5, and HLA-DPA1
5	Secretory-papillary FB	PTGDS, COL1A1, COL3A1, COL1A2, SFRP2, COL6A1, APCDD1, COL6A2, MMP2, and APOD
6	T cell	IL7R, CD52, PTPRC, CXCR4, HCST, CD69, CD48, FYB1, ACAP1, and LTB
7	Mesenchymal FB	ASPN, OGN, TNN, LUM, COCH, COL1A2, COL1A1, MXRA5, DCN, and COL3A1
8	Sebaceous gland	WFDC2, KRT19, CD24, CLDN4, KRT15, MMP7, SNORC, KRT7, SLPI, and PDE4B
9	Pericyte-2	ACTA2, TAGLN, RERGL, MYH11, MCAM, ADRA2A, PLN, NOTCH3, NDUFA4L2, and TPM2
10	Keratinocytes.undiff.-2	KRT15, FGFR3, KRT5, TP63, LGALS7B, COL17A1, COL7A1, KRT14, LGALS7, and CXADR
11	Hair follicle-1	KRT6A, LYPD3, S100P, DSG3, IGFL2-AS1, CDA, KRT6B, SERPINB5, KRT16, and DSC3
12	Sweat gland-1	PIP, AZGP1, KRT7, TSPAN8, KRT19, SCGB2A1, C5orf46, CEACAM6, PRR4, and CALML5
13	Secretory-reticular FB-1	PTN, SPON2, COMP, MFAP5, FGFBP2, RAMP1, COL14A1, ITGBL1, F2R, and IGFBP2
14	Sweat gland-2	AQP5, ROPN1B, S100A1, CLDN10, SNORC, ZG16B, PPP1R1B, KRT15, KRT7, and KRT18
15	VSMC	ACTG2, ACTA2, MYH11, CNN1, PCP4, PPP1R14A, MYLK, SAA1, TAGLN, and TPM2
16	Secretory-reticular FB-2	SFRP2, WISP2, THBS2, ADH1B, FBLN1, CLEC3B, MMP2, CTSK, TNXB, and CFD
17	Keratinocytes.diff.	KRT1, LGALS7B, KRTDAP, LY6D, DSG1, KRT2, CCL27, SERPINB2, SBSN, and DMKN
18	Sweat gland-3	PIP, SCGB1B2P, SCGB1D2, AZGP1, DCD, SCGB2A2, KRT7, MUCL1, KRT19, and KRT18
19	Mast cell	TPSB2, CTSG, CPA3, HPGD, TPSAB1, MS4A2, HDC, HPGDS, CMA1, and RHEX
20	Schwann cell	NRXN1, CDH19, SCN7A, S100B, PCSK2, MPZ, COL28A1, PMP2, XKR4, and PLP1
21	Hair follicle-2	KRT77, WFDC3, S100P, SPTSSB, KRT6A, KRT6B, KRT16, MMP7, CALML5, and HSPB8
22	Macrophages/DC	LYZ, AIF1, FCER1A, TYROBP, LST1, MS4A6A, FCER1G, MNDA, CLEC10A, and CPVL
23	Melanocyte	DCT, TYRP1, MLANA, PMEL, CRABP1, PCSK2, TYR, PLP1, KIT, and PHACTR1
24	Lymphatic EC	CCL21, TFF3, MMRN1, LYVE1, PKHD1L1, NR2F1, PROX1, CHRDL1, LMO2, and CAVIN2

### 3.3 Cell cluster and subset changes

**Figure 5:**
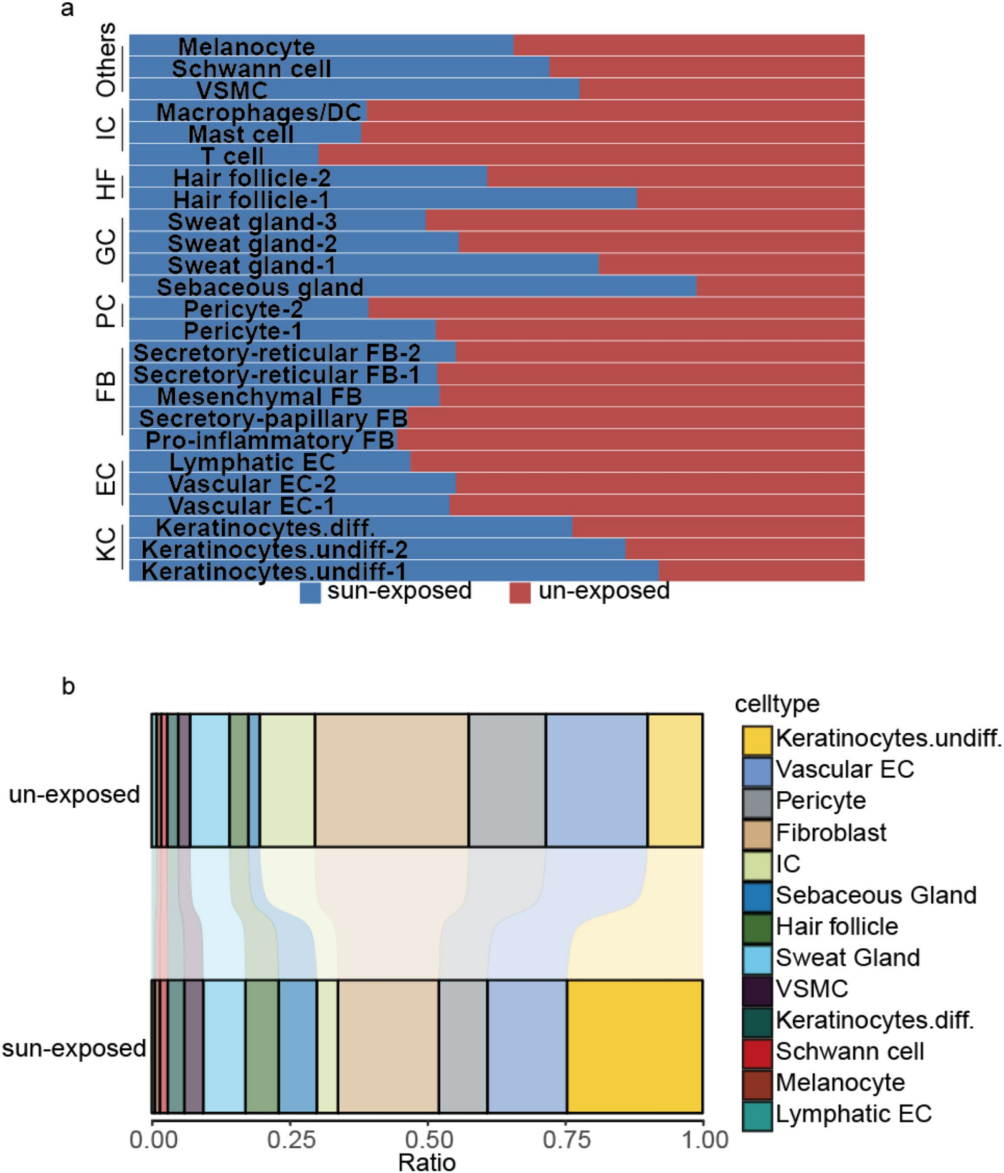
(a) Shows the percentage of the cell numbers in sun-exposed skin and unexposed skin for the 25 clusters, while (b) compares the cell subset ratio in sun-exposed skin with unexposed skin’s cell subsets. KC, keratinocytes; EC, endothelial cell; PC, pericyte; FB, fibroblast; HF, hair follicle cells; GC, gland cells, including sweat and sebaceous gland cells; IC, immune cell; VSMC, vascular smooth muscle cell.

The sample was divided into sun-exposed skin (4,704 cells) and unexposed skin (5,157 cells), and the percentages of each cluster were calculated in their respective groups after the cell annotation for each cluster (Figure 5). Compared to the unexposed skin, the increased clusters in the sun-exposed skin contained Keratinocytes.undiff-1 (#0), Keratinocytes.undiff-2 (#10), Keratinocytes.diff (#17), sebaceous gland cells (#8), sweat gland cells-1 (#12), hair follicle cells-1 (#11), VSMC (#15), Schwann cell (#20), and melanocyte (#23). Decreased clusters included vascular EC-1 (#1), pericyte-1 (#2), pro-inflammatory fibroblast (#3), vascular EC-2 (#4), secretory-papillary fibroblast (#5), T cell (#6), mesenchymal fibroblast (#7), pericyte-2 (#9), secretory-reticular fibroblast-1 (#13), sweat gland cells-2 (#14), secretory-reticular fibroblasts-2 (#16), sweat gland cells-3 (#18), mast cells (#19), hair follicle cells-2 (#21), macrophages/DC (#22), and lymphatic EC (#24) (Figures 2b, 5). In summary, compared to the unexposed skin, the increased cell subsets in the sun-exposed skin contained undifferentiated and differentiated keratinocytes, sebaceous gland cells, hair follicle cells, sweat gland cells, Schwann cells, vascular smooth muscle cells (VSMC), and melanocytes, while decreased cell subsets included vascular EC, pericytes, fibroblasts, immune cells (ICs), and lymphatic EC.

The observed differences in cell subsets between sun-exposed skin and unexposed skin highlight the significant impact of ultraviolet (UV) radiation on skin cellular composition and function. Understanding these changes provides insight into the mechanisms of photoaging and potential targets for therapeutic interventions.

### 3.4 Enrichment analysis for trending pathways

**Figure 6:**
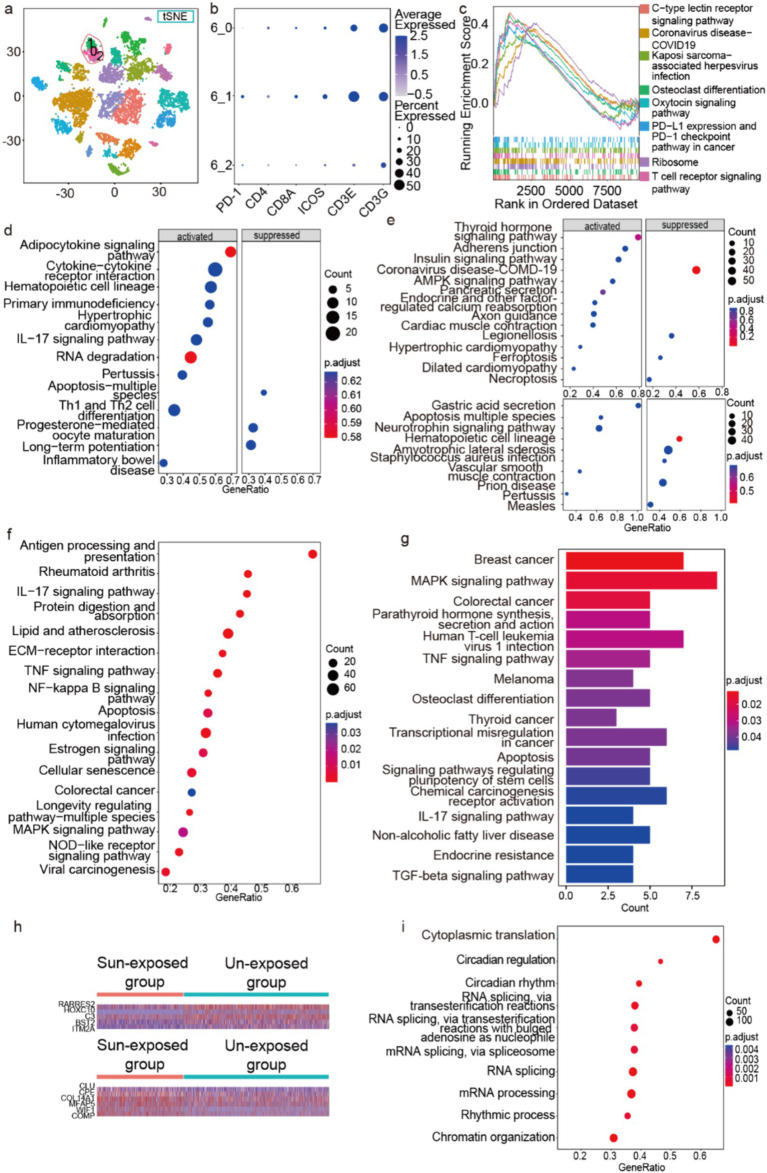
Enrichment analysis of immune cells, hair follicle cells, and Schwann cells. (a) Three subclusters of T cell *in situ* of the t-SNE picture. (b) Some marker genes of the three T-cell subclusters. (c,d) The GSEA of the DEGs obtained by the comparison of the sun-exposed and unexposed groups in T-cells’ subcluster 1 (c) and T cells (d). The GSEA enrichment of the DEGs is obtained by the comparison between sun-exposed and unexposed groups in mast cells ((e) above), macrophages/DC ((e) below), and fibroblasts (f). (g) The KEGG pathways of hair follicle cells’ upregulated genes in sun-exposed groups. (h) The heatmap of DEGs between fibroblasts’ sun-exposed and unexposed groups. (i) The GSEA enrichment of the DEGs from the comparison between sun-exposed and unexposed sebaceous gland cells.

As mentioned above, we have identified three immune cell clusters, which include T cells, mast cells, and macrophages/DC. To further analyze the functional role of immune cells in photoaging mechanisms, we first extracted the T-cell cluster, resulting in three subclusters identified through *in situ* clustering (Figure 6a), among which PDCD1 was significantly expressed only in subcluster 1 (Figure 6b). The GSEA analysis (minGSSize = 50, maxGSSize = 500) of the DEGs was obtained by the comparison of the sun-exposed and unexposed groups in subcluster 1 and showed 8 activated pathways including T-cell receptor signaling pathways, PD-L1 expression, and PD-1 checkpoint pathways in cancer (Figure 6c), which may indicate the relationship between photoaging and PD-1-PD-L1 pathway.

Compared to unexposed groups, T cells in the sun-exposed groups exhibited activated GSEA terms including primary immunodeficiency, IL-17 signaling pathway, and Th1 and Th2 cell differentiation (Figure 6d). These findings indicate that sun exposure may lead to immune dysfunction in T cells within the skin. Specifically, the activation of primary immunodeficiency and the IL-17 signaling pathway suggests functional impairments and abnormal immune responses in T cells following sun exposure. Overall, our results reveal an immunodeficient microenvironment and a more active Th cell function in the sun-exposed group. These findings not only provide new insights into the immune mechanisms underlying photoaging but also suggest potential targets for developing therapeutic strategies, particularly in modulating T-cell function and improving skin immune health.

Next, we performed pathway enrichment analysis on the DEGs obtained from the comparison between sun-exposed and unexposed groups in mast cells and macrophages/DC. We demonstrated that the enriched pathways are mainly related to diseases and hormone signaling pathways; conversely, ferroptosis and necroptosis pathways are suppressed in mast cells (Figure 6e above), while the apoptosis pathway was activated in macrophages/DC in the sun-exposed groups (Figure 6e below).

Subsequently, we used GSEA to explore the enriched pathways in the fibroblast clusters from the sun-exposed group. Compared to the unexposed group, the differentially expressed genes (DEGs) were primarily enriched in protein digestion and absorption pathways and ECM-receptor interaction pathways, suggesting accelerated ECM degradation after sun exposure (Figure 6f). In addition, the DEGs were enriched in pathways related to inflammation, cancer, aging, apoptosis, and several signaling pathways such as the IL-17 signaling pathway, TNF-alpha signaling pathway, NF-kappa B signaling pathway, MAPK signaling pathway, and NOD-like receptor signaling pathway. These DEGs and activated pathways could serve as potential therapeutic targets.

Moreover, the identification of differentially expressed genes (DEGs) in fibroblasts from sun-exposed skin highlights potential pathways and mechanisms that may contribute to the skin’s response to UV exposure. We identified five downregulated DEGs (ITM2A, BST2, C3, HOXC10, RARRES2) and six upregulated DEGs (COMP, WIF1, MFAP5, COL14A1, CPE, CLU) in the fibroblasts from the sun-exposed group compared to the unexposed group (logfc.threshold=0.25, *p*<0.05) (Figure 6h). ITM2A is involved in cell differentiation and proliferation (46, 47), the downregulation of ITM2A might suggest a reduction in fibroblast differentiation due to UV exposure, possibly contributing to impaired skin repair and regeneration. BST2 and C3 play a role in the immune response and acts as a restriction factor against viral infections (48, 49), reduced expression of which may indicate a compromised immune defense in sun-exposed skin, making it more susceptible to infections and inflammation. RARRES2 is known for its roles in apoptosis and inflammation regulation (50–52), and reduced levels of RARRES2 could lead to decreased apoptotic activity and altered inflammatory responses, potentially facilitating UV-induced skin damage and carcinogenesis. As for upregulated gene, COMP is involved in maintaining extracellular matrix structure and integrity (53). Article has shown that UVA irradiation of photoaged human dermal fibroblasts induced COMP expression at both the mRNA and protein levels, and the upregulation of COMP expression may contribute to the modulation of dermal extracellular matrix in the photoaging process (54). MFAP5 is associated with the formation of elastic fibers (41, 42) and COL14A1 (55) is involved in collagen fibril assembly. Their upregulation may suggest a compensatory mechanism to maintain or restore skin elasticity and structure following UV exposure. CLU (clusterin) is a multifunctional protein that plays roles in apoptosis, cell survival, and protection against oxidative stress (43–45). The upregulation of CLU may represent a protective response against UV-induced oxidative stress and apoptosis in fibroblasts. These DEGs may provide new therapeutic targets.

Finally, we investigated the human skin appendages, including hair follicle cells and sebaceous gland cells, and observed significant changes in gene expression following sun exposure. Compared to the unexposed skin, the upregulated genes in the hair follicle cells of the sun-exposed group were primarily enriched in KEGG pathways related to various cancers, such as breast cancer, colorectal cancer, melanoma, thyroid cancer, and transcriptional misregulation in cancer. In addition, these genes were enriched in the MAPK signaling pathway, TNF-alpha signaling pathway, TGF-*β* signaling pathway, IL-17 signaling pathway, and apoptosis pathway (Figure 6g). These findings suggest that sun exposure may increase the risk of carcinogenesis in hair follicle cells and activate various signaling pathways related to cellular stress and inflammation.

On the other hand, in the sebaceous gland cells of the sun-exposed group, the upregulated genes were mainly enriched in pathways related to cytoplasmic translation, circadian regulation of gene expression, rhythmic processes, mRNA processing, RNA splicing, mRNA splicing via spliceosome, RNA splicing via transesterification reactions with bulged adenosine as nucleophile, RNA splicing via transesterification reactions, circadian rhythm, and chromatin organization (Figure 6i). These results indicate that sun exposure leads to increased transcriptional and translational activity in sebaceous gland cells, which may be associated with cellular repair and adaptation to stress conditions.

These findings highlight the profound impact of sun exposure on the gene expression of skin appendages, revealing the molecular mechanisms involved in the response of hair follicles and sebaceous gland cells to UV damage. The gene changes in hair follicle cells may be closely related to the activation of cancer-associated pathways, while the gene changes in sebaceous gland cells indicate enhanced transcriptional and translational activities in response to sun damage. These results provide new insights into the molecular mechanisms of photoaging and may help in the development of preventive and therapeutic strategies against photoaging and skin cancer.

## 4 Discussion

Chronic UV irradiation can induce significant changes in various cell clusters within photoaged skin samples, including keratinocytes and other cell types; however, the specific process and cell-subtype changes remain unclear. In this study, we analyzed the single-cell transcriptomes from the same sun-exposed location (arm) and unexposed location (buttock) from the female healthy donor, which allowed us to minimize chronologic effects and provided insights into the differences of the cell proportions in human sun-exposed and unexposed skin.

Overall, 25 cell clusters and 12 skin cell types with significantly altered gene expression were identified in this study. By comparing the cell numbers in each cluster between sun-exposed and unexposed skin, we found that 9 cell clusters were more prominent in sun-exposed skin, while 16 cell clusters were more prevalent in unexposed skin. Notably, there was a significant increase in sebaceous gland cells, keratinocytes, VSMC, and melanocytes in sun-exposed skin, along with a decrease in fibroblasts, endothelial cells, pericytes, and immune cells compared to unexposed skin. This provided a preliminary basis to map the photoaging process in terms of cell subpopulation changes.

Upon analyzing the epidermal cell subtype ratio, we observed upregulation in both undifferentiated and differentiated keratinocytes in sun-exposed skin, with more pronounced changes in undifferentiated keratinocytes. This reveals that undifferentiated keratinocytes are more sensitive to UVR irradiation from the sun, suggesting a need for more focus on light protection for the epidermis.

In photoaged skin, collagen fibrils and elastin fibers, which constitute the majority of the dermal extracellular matrix, appear degraded and depleted (26). Based on previous studies, we subdivided the dermal fibroblasts into five subclusters and found that all subclusters decreased in number after UV radiation exposure, and the sun-exposed groups exhibited activated enriched pathways including protein digestion and absorption pathways and ECM-receptor interaction pathways, which could explain the dermal ECM changes of photoaged skin. This deepens our understanding of dermal changes in skin photoaging.

In our examination of skin appendage cell subtypes, we observed an increase of sebaceous gland cells in sun-exposed skin. KEGG pathway analysis indicated a more active gene expression process. Studies have shown that skin sebum is easily oxidized under repeated UVR exposure, leading to the production of several lipid photo-oxidative products, such as squalene peroxides. These products can be presented by CD1a molecules to T cells, which recognize the antigens via their T-cell receptors (TCRs), thereby accelerating the photoaging process (27). In sun-exposed hair follicles, the upregulated genes were enriched in MAPK, TNF-alpha, TGF-beta, IL − 17, and apoptosis signaling pathways, indicating that UVR can cause hair follicle cells apoptosis, primarily related to the MAPK signaling pathway, which controls balance of apoptosis (28–30), TNF-alpha signaling pathway, which participates in the cellular apoptosis (31, 32), and TGF-beta signaling pathway, which has been proven to be able to induce apoptosis (33, 34). This might be a protection reaction against damaged cells induced by solar light.

Previous studies have revealed that photoaging is conversely correlated with the capillary density and the pericyte-to-endothelial cell ratio (PC/EC ratio) of capillaries or venules, due to the relative and absolute loss of the PC (4). Our results are also in line with these observations of the relative and absolute loss of the PC.

Studies have also shown that sunlight can induce skin immune inflammatory reactions and immune microenvironment imbalance. Recent research has highlighted the important role of the PD-1 and PD-L1 pathways in the accumulation of senescent cells and inflammation associated with aging, due to their contribution to an immunosuppressive microenvironment (35). Our data also verify that in sun-exposed skin, PD-1-PD-L1 pathways are more activated in PD-L1+ T cells compared to unexposed skin, further demonstrating that solar radiation induces specific changes in the dermal immune microenvironment. Thus, our results provide a direction for further dermal pathophysiological and immunological investigation.

On the other hand, we observed the activation of the IL-17 signaling pathway in multiple cell types, including T cells, fibroblasts, and hair follicle cells, which is associated with inflammation. This activation is linked to senescence-related chronic low-level inflammation, known as inflammaging, which is closely connected to immunosenescence (36). Inflammaging leads to elevated levels of pro-inflammatory cytokines in circulation. Research has shown that senescent cells can activate the IL-17 signaling pathway (37). Studies have shown that IL-17 is a critical inflammatory mediator that plays a pivotal role in the development and progression of many inflammatory diseases such as osteoarthritis (38) and psoriasis (39). The research found that sweet pepper juices downregulated the expression of pro-inflammatory proteins such as IL-17, which leads to an overall reduction in UVB-induced skin damage (40).

Moreover, the DEGs identified in this study reveal potential targets for therapeutic intervention in sun-exposed skin. By modulating the expression of these genes, it may be possible to enhance skin repair, reduce photoaging, and prevent UV-induced skin damage. For instance, MFAP5 is associated with the formation of elastic fibers (41, 42), whose upregulation may suggest a compensatory mechanism to maintain or restore skin elasticity and structure following UV exposure. CLU is a multifunctional protein that plays roles in apoptosis, cell survival, and protection against oxidative stress (43–45), and the upregulation of CLU may represent a protective response against UV-induced oxidative stress and apoptosis in fibroblasts. Therapies aimed at increasing the expression of protective genes such as CLU and MFAP5 could offer new avenues for skin protection and rejuvenation.

However, this study does have some limitations. We recognize that anatomical differences and sample size are indeed important factors requiring further investigation. Therefore, in future research, we plan to include more skin samples from areas not exposed to sunlight (such as the inner upper arm) as controls to better differentiate the effects of sun exposure from those of anatomical location on skin transcriptome changes.

Taken together, our study has thus revealed the cell component changes in the photoaged skin samples. It provides novel insights into the repeated UVA-induced skin damage and pathology in the cellular subpopulation field and might help to further understand the delicate interplay of different cells in photoaged skin samples.

## Data Availability

The datasets presented in this study can be found in online repositories. The names of the repository/repositories and accession number(s) can be found at: https://www.ncbi.nlm.nih.gov/, GSE275491.
